# Diphyllobothriasis in a nine-year-old child in India: a case report

**DOI:** 10.1186/1752-1947-5-332

**Published:** 2011-07-29

**Authors:** KV Ramana, Sanjeev Rao, Moses Vinaykumar, M Krishnappa, Rajeshwar Reddy, Mohammed Sarfaraz, Vamshikrishna Kondle, MS Ratnamani, Ratna Rao

**Affiliations:** 1 Department of Microbiology, Prathima Institute of Medical Sciences, Nagunoor, Karimnagar, Andhrapradesh, India; 2 Department of Paediatrics, Prathima Institute of Medical Sciences, Nagunoor, Karimnagar, Andhrapradesh, India; 3 Department of Microbiology, Apollo Health City, Jubilee Hills, Hyderabad, India

## Abstract

**Introduction:**

The *Diphyllobothrium *genus belongs to the *Diphyllobothridea *order of tapeworms. *Diphyllobothrium *spp., which is commonly known as fish tapeworm, is generally transmitted in humans, but also in other species, such as bears, dogs, cats, foxes, and other terrestrial carnivores. Although worldwide in distribution, the original heartland of *Diphyllobothrium *spp. spreads across Scandinavia, northern Russia, and western Serbia. We report a rare case that occurred in India.

**Case presentation:**

A nine-year-old south Indian girl was brought to the casualty at the Prathima Institute of Medical Sciences with complaints of vomiting and loose stools that had started three days earlier. The vomit did not have a foul smell and contained no blood or mucus, but it did contain undigested food particles. The patient described a history of recurrent abdominal pain. She was a non-vegetarian and said she had a history of eating fish.

**Conclusion:**

The incidence of *Diphyllobothrium *spp. infection is infrequent in India. Since this is only the fourth reported case in India, and since the previously reported cases also involved observed pediatric patients, we emphasize the need for clinical microbiologists and pediatricians to suspect fish tapeworm infection and recommend epidemiological study of *Diphyllobothrium *spp. infection.

## Introduction

The *Diphyllobothrium *genus belongs to the *Diphyllobothridea *order of tapeworms. *Diphyllobothrium *spp., which are commonly known as fish tapeworms, are generally transmitted to humans [1]. Definitive first and second intermediary hosts of *Diphyllobothrium *spp. include humans, mammals and birds that eat fish, crustaceans, copepods, and fish. Salmonids, pike, perch, and burbot can act as secondary intermediate hosts of *Diphyllobothrium *spp. in freshwater ecosystems. Although worldwide in distribution, the original heartland of more frequent *Diphyllobothrium *spp. of the *Diphyllobothridea *order of tapeworms are spread across Scandinavia, northern Russia, and western Serbia [2].

## Case presentation

A nine-year-old south Indian girl was brought to the casualty at the Prathima Institute of Medical Sciences with complaints of vomiting and loose stools that had started three days earlier. The vomit did not have a foul smell and contained no blood or mucus, but it did contain undigested food particles. The patient described a history of recurrent abdominal pain. She was a non-vegetarian and said she had a history of eating fish. She had had a low-grade continuous fever for three days. Her loose stools were watery in consistency, were not foul smelling, and contained no blood or mucus, and the patient showed no signs of dehydration. She reported no history of similar complaints or any previous hospitalization. A general physical examination revealed the patient to be moderately built and dull looking, with a body temperature of 99°F, a pulse rate of 110 beats per minute, and a respiration rate of 22 breaths per minute. Her blood pressure recorded upon admittance to our hospital was 110/70 mmHg.

The hematological profile of the patient showed 9.3 g/dL hemoglobin, total red blood cell (RBC) count 3.82 RBC/mm^3^, a low hematocrit level of 27.6% (normal 37% to 47%), a below normal mean corpuscular volume of 72.3 μm^3^/RBC (normal 82 μm^3^/RBC to 92 μm^3^/RBC), a low mean corpuscular hemoglobin volume of 24.3 pg/cell (normal 27 pg/cell to 32 pg/cell), and a mean corpuscular hemoglobin concentration 33.6% (normal 32% to 36%). No eosinophilia (3%) was observed, and her erythrocyte sedimentation rate was found to be 10 mm per hour.

**Figure 1:**
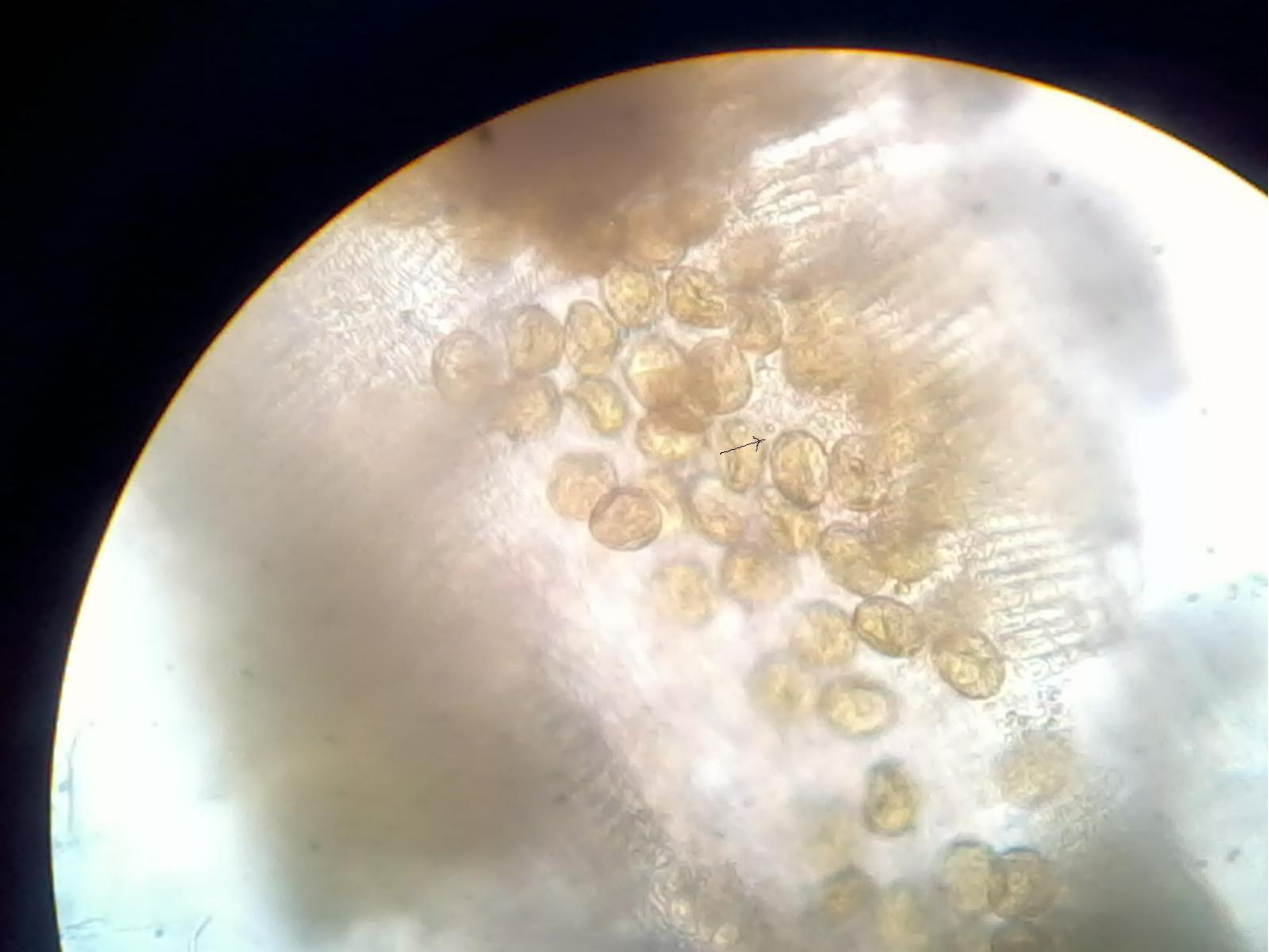
**Eggs of *Diphyllobothrium *spp**.

**Figure 2:**
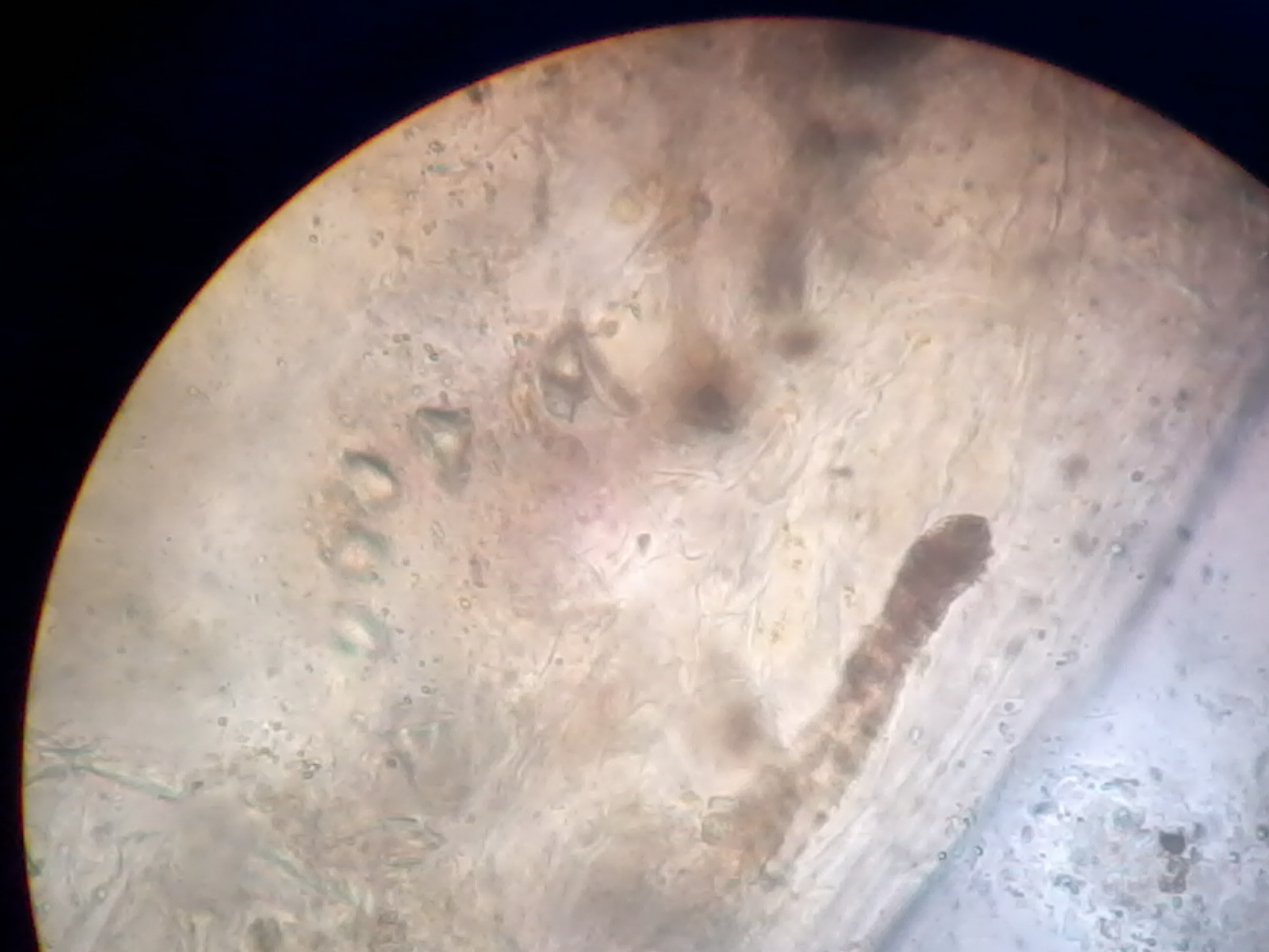
**Adult tapeworm showing scolex and segments**.

Stool samples obtained for ova and cyst examination were sent to the microbiology laboratory. Simultaneously, blood was sent for culture. Macroscopy of her stool revealed undigested material that was semi-formed but without any foul smell. White to creamish specks were observed in her stool, indicating the probable presence of tapeworms. A wet mount showed the presence of operculated eggs measuring 75 μm×40 μm (Figure 1). Characteristic broader than long segments of tapeworm were observed. On repeated wet mounts, scolex of the tapeworm along with gravid proglottids and a group of eggs were observed (Figure 2). On the basis of the morphology of the eggs with operculum and the presence of broader than long segments, as well as the scolex, the parasite was identified as *Diphyllobothrium *spp. The patient's blood culture was negative.

## Discussion

*Diphyllobothrium *genus belongs to the order *Diphyllobothridea*. There are six different *Diphyllobothrium *spp., including *Diphyllobothrium latum*, *Diphyllobothrium dendriticum*, *Diphyllobothrium klebanowski*, *Diphyllobothrium cordatum*, *Diphyllobothrium dalliae*, *Diphyllobothrium ursi*, and *Diphyllobothrium nihonkaiense*. *D. latum*, commonly referred to as "fish tapeworm," infects humans [3]. Diphyllobothriasis causes minimal local pathology, but is responsible for reduced vitamin B_12 _absorption and altered gut mobility [4]. The common symptoms include weakness, dizziness, salt craving, diarrhea, and abdominal discomfort. Diphyllobothriasis is associated with eating raw fish and is endemic to Serbia, Scandinavia, North America, Japan, and Chile, with more than 2% prevalence worldwide [2].

Although widespread in distribution, diphyllobothriasis is not often reported in India. Previous reports of fish tapeworm infection in India were from Pondicherry and Vellore, both of which are in southern India [5-7]. No cases in other parts of India have yet been recorded. In contrast to what was observed in previous studies, our patient showed no marked eosinophilia and presented with mild fever [5]. Anemia was established (9.3 g/dL), and the blood smear was normocytic and hypochromic in nature. This suggests that there was no marked vitamin B_12 _deficiency, which can lead to megaloblastic anemia in individuals infected with fish tapeworm. A detailed review of the previous literature revealed that only three previous cases in India have been reported, and in both cases, the infections were in pediatric patients, in contrast to what has been observed in recent Korean cases of diphyllobothriasis, which involved middle-aged individuals [8].

## Conclusion

Our findings suggest the probable undiagnosed parasite manifestation in pediatric patients. We therefore recommend epidemiological studies of fish tapeworm manifestation in pediatric patients, as the infections, if undiagnosed or underreported, can lead to considerable morbidity.

## Consent

Written informed consent was obtained from the patient's next-of-kin for publication of this case report and any accompanying images. A copy of the written consent is available for review by the Editor-in-Chief of this journal.

## Competing interests

The authors declare that they have no competing interests.

## Authors' contributions

KVR analyzed and interpreted the patient data regarding the *Diphyllobothrium latum *infection and performed the parasite identification. KVR and DSR were major contributors in writing the manuscript. BVM, MK, and RR all contributed to writing the manuscript. MSN and KV evaluated the patient clinically. All authors read and approved the final manuscript.
